# Exploring the types of approaches used by community food organisations and their role in food insecurity prevention: a mixed-methods study

**DOI:** 10.1136/bmjopen-2026-122055

**Published:** 2026-10-22

**Authors:** Maria Bryant, Giorgia Previdoli, Rachel Benchekroun, Wendy Burton, Maddy Power, Philip Hadley, Ariadne Kapetanaki, Bob Doherty, Claire Cameron, Kate Pickett, Laura Sheard

**Affiliations:** 1 Hull York Medical School, University of York, York, UK; 2 Department of Health Sciences, University of York, York, UK; 3 Social Research Institute, University College London, London, UK; 4 Department of Public Health, Environments and Society, London School of Hygiene and Tropical Medicine Centre of Global Change and Health, London, UK; 5 School for Business and Society, University of York, York, UK; 6 School of Health Sciences, University of Manchester - The Victoria University of Manchester Campus, Manchester, UK

**Keywords:** Food Insecurity, PUBLIC HEALTH, Family

## Abstract

**Objectives:**

Community food organisations are local food infrastructures that respond to household food insecurity (though they may be nationally managed). They are diverse in their approaches and include, among others, emergency food distribution, food pantries, community kitchens and food banks. Many provide additional support, including welfare advice, employment support, food vouchers and signposting to helpful resources. We investigated what approaches used by community food (aid) organisations are most likely to help prevent the need for emergency food for families in two multicultural areas in the North and South of England.

**Design:**

A longitudinal mixed-method research programme, including systems mapping, survey and workshops, semi-structured qualitative interviews and a multiple-method longitudinal qualitative study (ethnography, go-along interviews and visual study).

**Setting:**

Community settings, including ethnographies in nine community food organisations across two local government areas. Data collection took place from July 2023 to July 2025.

**Participants:**

89 community food organisations and ~160 representatives took part across work packages, including workshops (n=82 local/national representatives), survey (n=89 organisations), ethnography (n=9 organisations); interviews (n=36 funders, coordinators, managers and volunteers); longitudinal interviews (n=40 families with lived experience of food insecurity) and visual study (n=36 families).

**Results:**

Community food organisations are part of a complex, fragile food system, with high dependency on surplus food, volunteers and external funding. Although many aim to address underlying issues, food insecurity prevention is often not possible in practice, largely due to operational tensions (space, capacity and funding constraints) and wider financial and political constraints

**Conclusions:**

No single model of delivery within community food organisations appears more effective at preventing the need for emergency food. Instead, sufficient space, time and resources to address underlying needs and staff approachability are most likely to make a difference. However, any influence organisations play to reduce food insecurity would be minimal without tackling poverty as an underlying cause.

**STRENGTHS AND LIMITATIONS OF THIS STUDY**

- This research included a large programme of work using multiple methods to explore roles played by community food organisations from a systems perspective, considering wider contextual factors.
- Data were collected from 160 actors across the system, including organisations, professional representatives, volunteers and members of the public with lived experience; providing an understanding of the system and the roles and dependencies of community food organisations within it.
- In-depth ethnography over 14 months was conducted using a Researcher in Residence approach, with three researchers in nine varied community food organisations.
- Research included meaningful involvement and engagement of families with lived experience, including ongoing study support and co-creation of a photo exhibition, formed from an embedded visual study.
- Research did not include a quantitative evaluation of the effectiveness of approaches, but captured a rich account of the influence of organisations and the potential of their approaches to support families with food insecurity.

## Introduction

Food security is defined as ‘having physical, social, and economic access to sufficient, safe, and nutritious food’ by the WHO.^1^ In contrast, food insecurity has been consistently linked to poor diet quality and increased risk of diet related disease, including cardiovascular disease, obesity and poor mental health.^2^ There was a notable increase in food insecurity during the COVID-19 pandemic that has persisted, with approximately one-third of the world’s population still unable to access the healthy food they need.^1^ In the UK, data collected between 2023 and 2024 indicate that 10% of all households were food insecure,^3^ with higher rates in families with children (16%). Families with children were also more likely to use food banks (6%) than households without children (3%). Data from Trussell (the UK’s largest formal food bank operator, a registered charity in England and Wales (1110522) and Scotland (SC044246)) suggests that their provision of food parcels increased by over 50% between 2020 and 2025 and that two-thirds of parcels were given to families with children.^4^

There has also been growth in the availability of other, less formal forms of community food support over the same period. Such food aid organisations (hereon termed ‘community food organisations’) are often local food infrastructures that attempt to respond to household food insecurity (though can be locally or nationally managed). Community food organisations include a diversity of models (ie, mode of delivery) and components (ie, activities and actions). More broadly speaking, they include provision of ‘emergency’ assistance (eg, food banks, food/parcel delivery services, surplus food redistribution and soup runs), ‘non-emergency’ assistance with food (eg, community cafes, community supermarkets (offering low cost or pay-as-you-feel food) and food pantries) and wider social or welfare support. For many organisations, food provision is largely dependent on donations and therefore varies in terms of the offer, its nutritional value and its cultural and/or religious acceptability.^5^ This can be ‘topped up’ by financial support from national or local governments, though again, this is not consistently implemented between or within areas.

Community food organisations use varying approaches for supporting different populations. In theory, they are available to anyone who needs them. However, some community food organisations, particularly food banks, operate on a referral basis from professionals or organisations such as social workers, health visitors, schools or Citizens Advice. Such referrals can be instigated from the person in need (ie, seeking support from Citizens Advice) or identified by a professional. Other community food organisations, particularly ‘non-emergency’ provision and organisations which are not part of a wider network, tend to provide support without the need for referrals (including those supported by local governments or community ownership funds). However, they might still apply eligibility criteria, for example, requesting to provide evidence of benefit receipt and/or an address (for those with geographical restrictions). Some also only support specific populations (refugees for example).

Community food organisations include both well-established organisations and those that are set up responsively. They are available in a wide variety of settings, including schools, religious settings, community halls and other community hubs. Many areas have central Food Hubs, where operations are coordinated across multiple settings (eg, sourcing, storing and distributing surplus food).^6^ Previous research has argued that more ‘traditional’ food banking approaches (which have focused on emergency food provision) do not offer long-term solutions to food insecurity and the evidence on their benefits in terms of health and welfare is inconsistent.^7^ While there is no doubt that emergency food is essential during times of crisis, many organisations have been trying to reduce the need to rely on a food-only approach by providing secondary prevention support, that is, services such as providing financial support and signposting to additional resources (sometimes termed ‘wraparound’ support).

For example, many community food organisations (including food banks, eg,^8^) have diversified their approach; some combining emergency food with the provision of long-term support (such as welfare and/or employment advice), some providing low-cost/high quality food (that can be chosen) and a small minority, piloting a cash-first approach (delivering support through cash-payment rather than food, whenever possible).^9–11^ There is limited understanding of the degree to which these approaches support people, and families in particular, to become food secure.^12^

While franchised food banks such as Trussell have been widely studied (eg,^11 13^), there has been less research about the roles and potential impact of other community food organisation approaches, including their potential to reduce reliance on emergency food and their inclusivity and accessibility. There is limited information about the use of community food organisations among different ethnic groups, but there is some agreement that people from ethnic minority groups may experience particular structural barriers in accessing it.^14 15^ Research has also shown limited ability to consistently provide culturally appropriate food.^13^ This is particularly pertinent as food insecurity is greater in some ethnic groups in the UK, particularly black African, black Caribbean and black British (22%), Mixed and multiple ethnic groups (20%) and Arab households (21%), compared with white households (8%).^3 16^

To fill gaps in evidence, we conducted a programme of work among multiethnic populations to explore the community food organisation components/activities which are described as providing primary prevention of food insecurity (eg, through supporting educational or employment opportunities), secondary prevention (eg, through social security advice) and the provision of tertiary support (ie, the mitigation of the immediate impact of food insecurity via provision of food).^17 18^ We then considered the degree to which these community food organisation operation models or components could be most successful at preventing food insecurity. This was intended to generate rich evidence to inform culturally appropriate, sustainable community-based policies which could be applied to other diverse populations.^18^ Given the need to effectively target resources to support those in greatest need, this evidence is particularly crucial to both local and national decision-makers.

We asked (1) What role do community food organisations play among other factors that influence food insecurity, including the types of approaches used to prevent the need for emergency food aid; (2) What does the community food support system look like, including key structural and economic domains, how do they relate to one another and what interdependencies exist; (3) In what ways do different community food organisation models and components provide primary, secondary or tertiary prevention of food insecurity; and (4) What impact do these have on the food security status of families?

## Methods

### Design

We conducted a mixed-methods study, integrating (1) systems mapping (surveys with community food organisations to understand organisational structures and objectives, and workshops and interviews to build a picture of the community food organisation system, its dependencies, relationships and operational pathways) and (2) longitudinal qualitative research (ethnography within community food organisations using a Researcher in Residence model, ’go-along’ interviews with families experiencing food insecurity (see ‘Go-along interviews’ below) and a visual study with families) (table 1).^18^ We took a complexity science/systems thinking approach, underpinned by Hawe’s (2009) ‘theorising interventions as events in systems’^19^ to understand which community food organisation models, components and prevention approaches might be best able to reduce dependence on forms of emergency food aid.

**Table 1:** Methodological approaches across the programme of work

Methodology	Sample size	Data collection period	Primary objective
Survey	89 community food organisations (27 from Tower Hamlets, 62 from Bradford)	July 2023 to January 2024	To understand organisational structures, objectives, models, components and localities.
Systems mapping workshops (exploratory, confirmatory and public)	6 workshops (n=42 exploratory, n=15 confirmatory and n=25 members of the public)	September 2023 to December 2023	To build a picture of the community food organisation system, its dependencies, relationships and operational pathways from stakeholder and public perspectives.
Systems mapping semi-structured interviews with community food organisation and national representatives	36 interviews (24 community representatives; 12 national representatives)	January 2024 to March 2024	To provide more clarity and granularity of the community food organisation system, its dependencies, relationships and operational pathways.
Ethnography (embedded researcher in residence model)	3 researchers in 9 community food organisations	January 2024 to April 2025	To carry out participant observation within community food organisations in order to understand everyday practices and families’ experiences
Go-along interviews (three waves)	40 families (30 community food organisation users, 10 non-users)	June 2024 to July 2025	To learn from families with experience of food insecurity about the role played (or not) by community food organisations over a 12-month period.
Visual study (photo elicitation)	36 families (over 300 photos)	June 2024 to July 2025	To share families’ experiences related to food and help with food via photo elicitation.

All aspects of the research involved support from a project specific public advisory panel. This included people with lived experience of food insecurity from varied backgrounds. In addition to supporting the research design and implementation processes, the group allowed us to carefully consider the sensitivities of the topic and the potential vulnerabilities of the population involved, for example, mitigation strategies to counterbalance imbalance of power between researchers and participants.

### Setting

Research was carried out in two areas within the UK; Bradford (a city in Northern England) and Tower Hamlets (a Borough of London). Both areas are ethnically diverse with high levels of poverty. In Bradford, approximately 62% of residents identified as ‘White’ and around 32% identified as ‘Asian or Asian British’ in the most recent census.^20^ In Tower Hamlets, 44% and 39% of residents identified their ethnic group within the ‘Asian, Asian British or Asian Welsh’ or ‘White’ categories, respectively. In both local authorities, around half of children live in poverty after housing costs^21^ compared with a UK average of 31%.^22^ In Tower Hamlets, in 2021, around two-thirds of low-income parents reported that they had insufficient money for food.^23^

### Population

All aspects of research involved representatives across the community food organisation system, including organisational representatives (eg, managers, volunteers, staff) and representatives from organisations outside of the immediate community food organisation network that were considered to play a role in the local systems (eg, local government, surplus food redistribution charities, national experts) and families with children experiencing food insecurity (both those using and not using community food organisations).

Community food organisations that were invited to take part in the ethnography were sampled using findings of the systems mapping workshops, where we intended to recruit a diverse range of organisations, including both franchised and under-researched organisations (eg, those responsive to local populations (eg, in terms of religion or ethnicity), or have developed organically to meet local needs). Sampling also considered the type of support offered, to include a range of those providing ‘primary’ support (ie, aiming to alleviate food insecurity via support such as employment opportunities), ‘secondary’ support (ie, those mediating the impact of poverty through welfare guidance) and ‘tertiary’ support (ie, mitigating short term impact via emergency or low cost food). We initially proposed ethnographic research across five community food organisations. The sample size of the community food organisations was increased from an initial five sites to nine sites in order to capture emergent variation during preparatory work and relationship building on the ground.

To recruit families for go-along interviews, we purposively recruited a range of families to ensure diversity in terms of ethnicity, religion, disability and family size (including single parents). We planned to recruit 35 families who had one or more children under 18 and a lived experience of food insecurity to take part in three go-along interviews over 12 months, to follow people’s everyday lives to understand their food insecurity status in relation to supportive strategies (including community food organisation models and components). Of these, 20 families were planned to be recruited from the community food organisations within which the ethnographies were being conducted, with a further 10 recruited who did not use community food organisations. Our initial sample size of 35 families at wave was designed to anticipate a relatively high rate of attrition in longitudinal interviewing between wave one and wave three.

### Data collection

#### Surveys

Surveys were distributed to all community food organisations in the two localities (July 23 to January 24) to identify what different organisational models and components were adopted and to explore how accessible and inclusive the services were. These surveys also sought to explore the relationships between organisations and understand what they depended on to operate. Existing lists of organisations (that were available through previous networking) or publicly accessible online were used to share surveys via an email link. A snowballing approach was used thereafter to enable organisations who were approached to share with other organisations. The survey was also used to recruit organisations to take part in system mapping workshops at both localities.

#### Systems mapping

System mapping methods were applied to explore and conceptualise the community food organisation system, to provide an understanding of key relationships and dependencies between organisations and to identify the position of community food organisation systems within broader food and welfare systems. This work has been published elsewhere.^24^ In brief, two exploratory (n=42) and two confirmatory (n-15) workshops were conducted with a range of representatives across the system (staff and volunteers from community food organisations (eg, food banks, food pantries, food hubs), individuals from public sector organisations, health and education sectors and food redistribution organisations). Over the same period (September 23 to March 24), two additional workshops were held with members of the public with a lived experience of food insecurity (n=25) to seek their perspectives on the ways that community food organisations support families with children.

To add further clarity and depth across a wider area, interviews with 24 community food organisation representatives from food banks, pantries, community kitchens, food hubs, community food organisation networks, anti-poverty steering groups, food redistribution organisations and local government were conducted (January to March 24), as well as 12 interviews with national representatives (eg, national network coordinators, directors of national charities, campaign leaders, food sector professionals) (see online supplemental file 1 for interview guides). Using these data, a ‘concept map’ was developed by the research team and a series of workshops and interviews were then hosted to refine and agree a final systems map.^24^

10.1136/bmjopen-2026-122055.supp1 Supplementary data

#### Ethnography

Three experienced researchers (GP, RB and WB) conducted an ethnography within nine community food organisations over 14 months (January 2024 to April 2025). None of the researchers had personal experience of food insecurity; one was an experienced public health nutritionist. Organisations were purposively chosen to represent a diverse range of characteristics, ensuring that we included under-researched forms of community food organisations (eg, those which are responsive to local populations (eg, in terms of religion or ethnicity), or that developed organically to meet local needs). This was important, as evidence suggested that some of the most vulnerable people in need of food aid never present to a formal food aid provider.^25^ Given the sensitivity of the topic (experiences of food insecurity and help with food)^26^ and the precarious life conditions of many families, we chose a methodology that was deep, immersive and appropriate to build mutual trust. Thus, a ‘researcher in residence’ approach was used, whereby researchers volunteered in organisations in order to embed themselves in the daily life of each organisation and gain the trust of other volunteers and users.^27^ After 3 months, the researchers began to approach potential participants to take part in go-along interviews and a visual study.

#### Longitudinal go-along interviews

Families were invited to take part in three ‘go along’ interviews over approximately 12 months, each lasting from about an hour to half a day each. In contrast to static interviews in a single location, ‘go along’ involved the researcher going (travelling) alongside the participant as they went about their everyday usual activities with researcher and participant talking along the way.^28^ An open-ended interviewing style was used, following people’s everyday lives to understand their experiences of food insecurity and the potential role of community food organisation models and components, as well as wider factors. All families were invited to meet with a researcher three times, regardless of whether they were still accessing food support or not. We wanted to understand whether they had moved out of food insecurity and the complex factors that may have enabled this (including the potential role of community food organisations).

#### Participatory visual study

All families who were recruited to take part in the go-along interviews were invited to take part in a participatory visual study, whereby they were asked to take photographs of things that they felt were important to them in relation to food. Participants shared their photos (and associated thoughts) across 12 months using research app Indeemo, and where this was not possible, using WhatsApp. In addition to acting as a catalyst for discussion in the go-along interviews^29^ the photographs allowed us to develop a powerful resource to stimulate community and policy discussion through photography exhibitions. An interactive workshop was organised for 25 participants who agreed to take part, to facilitate the development of photography skills to explore the potential of photography as a form of expression and to empower participants to share their own experiences and desire for ‘change’ through visual methods. Resulting photographs and quotes were then organised in themes, in an iterative co-curation process which involved participant families, researchers and public members and was facilitated by the professional photographer.

### Analysis

#### Systems understanding

Transcripts and drawings from exploratory workshops and survey responses were reviewed by four researchers (GP, RB, WB and MP) to identify system elements (eg, actors, activities). These elements (nodes) were entered into an adjacency matrix^24^ in Excel for each locality, using binary variables (‘1’/‘0’) to denote relationships. Kumu software (Kumu 2011–2024) was used to convert the matrices into draft maps, which the research team and community food organisation representatives iteratively refined during confirmatory workshops. Transcripts from these sessions and stakeholder interviews were analysed to add further nodes and connections.

In the final stage of the process, a map of a local community food organisation system was developed by merging the two local-specific maps to generate a more generalisable representation of local community food organisation systems in England. First, one researcher compared the two local maps to identify similarities and differences (WB), then the nodes were thematically grouped to simplify. Using an adjacency matrix and Kumu software, two researchers produced the final generic map (WB and GP), which was then reviewed by the wider team.

In addition to the systems map, we created a comprehensive conceptual typology map of community food organisations to illustrate models, activities, relationships and challenges within the community food support sector (led by PH). This built on the systems map by integrating new elements that were identified by national representatives both at workshops and in interviews. Following a similar process to the previous stages, the researcher used Kumu software to produce a typology map.^6^

#### Ethnography

Researchers wrote fieldnotes after each research in residence session. The notes were read several times and then ‘pen portraits’^30^ were produced for each setting, following a commonly agreed structure (Location; Set up and timing; What is on offer; Funding; The people who run it; The people who use it; Everyday practices: Interactions in the setting). Pen portraits were chosen because they facilitate the organisation of large datasets generated across multiple points in time into focused narrative diachronic accounts.

#### Interviews

Interviews conducted with national representatives were combined with data from the systems workshops transcripts and analysed through a thematic analysis process to explore how participants navigate and represent key paradoxes and dilemmas in the food charity sector, using Actor-Network theory as a sensitising tool.^31^

Go-along interviews with families were recorded and transcribed with participants’ permission and fully anonymised (by GP, WB, RB and LS). Notes were also taken of any encounter with participants (eg, if interviews were conducted while participants were collecting a food parcel). The transcripts were read several times and ‘pen portraits’ were produced for each participant, following a commonly agreed structure (life synopsis and changes in circumstances over the 12 months, access to food, access to support, relationship with community food organisations). Field researchers met weekly to reflectively discuss their observations and share mutual support; the lead of the qualitative longitudinal study joined the meetings monthly (LS). The first wave of go-along interviews was designed to be open-ended and participant-led to build a relationship between researcher and participant and to understand what issues were important to participants. For waves 2 and 3 of the go-along interviews, we designed topic guides which included topics for the researchers to ask participants about, but remained participant-led (online supplemental file 2).

10.1136/bmjopen-2026-122055.supp2 Supplementary data

### Reflexivity

The ethnographic researchers reflected throughout the research process (from fieldwork to analysis and writing) on how different aspects of their identities influenced how they interacted and built relationships with participants, how data was generated and how they analysed it. They regularly reflected as a team on how their gender, motherhood status, migration/citizenship statuses, religious beliefs and researcher roles shaped the research, and reflexively discussed their own assumptions and perspectives. For example, they felt that being mothers themselves helped to connect with participants, all of whom had children under 18.

Participants’ willingness to discuss their experiences will have varied depending on their circumstances at the time as well as the rapport they felt with the researchers.

### Data integration

The interviews and workshop transcripts from the systems mapping were coded line by line. The field notes from ethnographic observations and the interview transcripts with parents were distilled into pen portraits. The analytic integration was led with a focus on answering our core funded research questions. These were: (1) what does the community food support system look like across two cities? (2) can community food organisations support people beyond and, if so, how? and (3) do community food organisations have a role in preventing food insecurity for families and, if so, in what way?

We followed an iterative process to enhance rigour: first, the lead author engaged with the data and other material such as team presentations then second, the lead author engaged in reflexive conversations with the researchers who collected the data in the field. Finally, the lead author and the researchers who had collected the data drafted sections of findings under the headings of the three core research questions and shared the content with the wider team for feedback and suggestions.

### Patient and public involvement

We worked with 10 members of the public with direct and/or indirect lived experiences of food insecurity in the set up and delivery of all activities outlined in this paper. Membership was a diverse group in terms of ethnic background and lived experiences. In addition to regular meetings to provide meaningful guidance and oversight, members of this group provided specific input to the study design (including terminology, website branding and promotion animation), setting up recruitment (co-designing leaflets and screening forms), ensuring representation during recruitment (highlighting gaps that resulted in targeted approaches), co-designing outputs, including policy briefs and publications (noting when findings did or did not represent their own experiences), co-curation of the photo-exhibit and active participation at exhibitions. Participants and patient and public involvement members were invited to celebratory events for the launch of the photo exhibition where the research findings were presented and discussed. Members of the public also took part in a final meeting where key findings from the study were discussed.

## Results

Table 1 provides an account of the organisations and people that took part in the full programme of research. In brief, surveys were completed by 89 community food organisations (62 in the Northern site and 27 in the London-based site). We recruited representatives from across a wide and diverse range of the community food organisations and linked organisations (eg, in health and education) for workshops and interviews, including food banks and food pantries based in different settings (schools, faith-based, community centres), with/without wrap-around services (such as benefit advice), community kitchens, sport/cultural initiatives helping members with food, outreach teams, cooperatives, surplus food hubs, national and local networks. 40 families took part in the go-along interviews (5 more than planned) including 30 who had experience of using community food organisations and 10 who were food insecure but did not use community food organisations. Of these, 30 took part in three interviews, 31 took part in two interviews and 9 participants were only interviewed once. Of the 40 participants who agreed to take part, 36 also agreed to take part in the visual study, where food preparation, meals and food prices were most commonly photographed.

### The community food support system: complex and fragile

Our research revealed a complex food system on which many families rely on a regular basis rather than in discrete, emergency situations. Findings from the survey, mapping and interviews highlighted a number of features and operations. These are highlighted in table 2, but are by no means exhaustive, with many organisations offering a wide range of support.

**Table 2:** Activities and operational models of community food organisations

Organisation type	Key features	Funding and operations
Food banks	Provision of free, emergency food parcels. Often include non-food items (toiletries, baby supplies). Operate via referral systems or ‘open access’ with criteria. Some offer choice over items.	Run by voluntary/community sector, often faith-based. May be part of national networks (Trussell, IFAN). Funded by donations (public, corporate), local authorities. Volunteers are the main workforce but there is also increased professionalisation of leading paid roles.
Food pantries/social supermarkets	Provision of low-cost food, typically through a membership model requiring a weekly/monthly contribution. Emphasis on choice and dignity, offering fresh and ambient food.	Often run by existing charities. May be part of networks (Your Local Pantry, Affordable Food Clubs) funded partially by member contributions and partially by local authority or charity grants. Primarily volunteer-run, but leaders tend to be employed
Community centres and local charities	Often host other forms of support like food banks, pantries or community cafes. Provide low-cost or free community meals.	Variable. Often funded through grants, local authority contracts and public donations.
Soup/street kitchens	Provide hot meals in public spaces, primarily for street-living populations. Some also distribute food parcels without referrals.	Run by established charities or informal community groups. Links to local businesses for food donations. May have connections to local authority outreach and drug/alcohol services.
Community cafes and lunch clubs	Offer low-cost not for profit meals, often where other services are available. Serve specific geographical areas or communities.	Hosted by community organisations. Funding from grants and meal sales.
Community kitchens	Facilitate cooking sessions targeting groups without cooking facilities (often including provision of meals thereafter).	Run by charities or community centres.
Faith-based organisations	Churches, mosques, gurdwaras, temples and synagogues run food banks, pantries and offer free meals and advice.	Integral part of their community mission. May be part of wider faith-based or secular networks.
Food sharing apps and platforms	Digital platforms connecting users to share surplus food. (eg, Olio, Neighbourly, FoodShare and Too Good To Go). Primarily focused on waste reduction.	Technology-based platforms. May or may not be used by people experiencing food insecurity.
Food co-operatives	Member-owned organisations that buy food and household items in bulk to sell to members at affordable prices. Emphasis on self-organising and member control.	Funded by member contributions and support from co-operative networks (eg, Cooperation Town).
Educational institutions (including youth, sport and art-based initiatives)	Schools provide Free School Meals and breakfast clubs. They can also run food banks, food pantries or free collection tables. Universities may have hardship funds and on-campus food banks. Youth sport clubs or theatre groups may provide emergency food to members.	Government-funded programmes (schools), partnership with local charities/networks to host food banks or pantries (schools) or internal university funds.
Local food growing groups	Urban/city farms and community gardens. May have links to local food support networks.	Community-led, grant-funded, can be in partnership with council (community run allotments).

IFAN, Independent Food Aid Network.

Our mapping activities identified 67 system nodes across five domains that describe the context and delivery of community food operations including: (1) Economic context (highlighting the reliance on external funding streams and the systemic economic drivers of food insecurity, such as the high cost of living and the lack of affordable housing); (2) Food supply (outlining food procurement methods, particularly private donations, supermarkets and redistribution surplus); (3) Supporting organisations (including public and non-profit entities that host or support community food organisation and that provide broader community services, such as advice, language and digital support); (4) Community food organisation models and components (encompassing different models of support offered to people, such as differing food provision models (eg, food banks, food pantries, free/low-cost food) as well as support ‘beyond food’ (eg, welfare advice)); and (5) Access routes (including formal and informal referral pathways). These have been described elsewhere.^24^ This work highlighted not only the diverse nature of community food organisations, but also that they represent a constantly adapting system which is highly complex and fragile, with a number of interconnections and dependencies. Key dependencies identified included funding streams (eg, local government funding, charitable grants and private donations), surplus food redistribution and volunteer labour. For example, pathways within the system map identified that most organisations were dependent at that time (directly or indirectly) on central government’s wider ‘cost of living’ support, which, at the time, was known as the ‘Household Support Fund’.^32^ Representatives across the system told us that this funding was a critical source to operate and the impact of its potential removal was observed in practice during the ethnographic fieldwork, with organisations withholding food purchasing in times of uncertainty.^33^

The dependency on volunteers was also witnessed and shared across the programme, with data from an interview with a national campaigns officer noting that many community food organisations are in ‘really precarious situations’, being ‘reliant on volunteers…often one or two people’ (interview with campaign officer, National Organisation 13). Surplus, redistributed food was another key dependency within the system, without which a large proportion of the system would effectively ‘crash’ should it be removed without an alternative strategy. While some participants argued that surplus donations are an efficient way to prevent food waste and cut cost of waste transport, others argued that, when the quality is low, the cost and effort to dispose of waste is shifted from businesses to community food organisations and, in some cases, to service users. The commercialisation of surplus food distribution, also, with substantial investment into infrastructure expansion, contributed to the institutionalisation of the sector. Interview data from representatives across the system also raised concerns about the quality, diversity and cultural suitability of surplus food:

And is that shop sustainable if it’s dependent on surplus food which is being depleted, just as more and more pantries are evolving? Volunteer labour, you know, it’s limited, charitable grants are limited […] it’s alluring to think of them as more dignified and more, you know, providing more choice, but there is a limit to that dignity and there is a limit to that choice, and we can’t be fooled (interview with network director, national organisation 9).

### Are community food organisations able to support people beyond food and what does this type of support look like?

Findings from across the programme of research suggested that organisations have a number of objectives beyond emergency food aid, including provision of support in accessing social security, financial advice, employment support, setting up savings via credit unions, facilitating access to fuel grants, onward referrals (eg, to a baby bank), reducing isolation, providing cooking spaces (and/or classes) and growing food. However, pressures were faced on a daily basis, because of funding, physical limitations, capacity and complexity of users’ needs. This means that, in many cases, efforts to address issues underlying food insecurity had limited visible impact. Of the 30 families who took part in all three go-along interviews, just 1 highlighted that they had improved living conditions and income, in part because of the support from the community food organisation to maximise benefits and reduce energy bills.

One of the issues we observed was that, even when advice and additional services were present in principle, families accessing the food provision were not fully aware of what else they could expect to find.

the pantry is “you take your food then you go – whatever I am giving you, you should be grateful”, so this is the way it’s going on – so when you have the time to talk with us? […] If your company’s main model is helping vulnerable people, not only food-wise, it should be more accessible. […] You know, you could tell the families that [these are the places you can go/support available], then I can go to those places. But you didn’t tell me, but you postering that you give people the opportunity, you show them that this is happening, this is happening, but you are only showing me one thing… It’s like a big department, superstore, you just show me the food aisle, but there is a bakery, there is toiletries, there is home… you didn’t show me, but you are signpostering that you show me all of this. Because I am busy with the one aisle, I’m thinking there is no aisle behind there. If I don’t know, how can I explore the other aisles? (parent 1, interview 2)

However, in the majority of cases, the need to slow down, to be able to ask people about their wider needs, sometimes conflicted with the transactional management style required to efficiently deliver predictable, high volumes of food in relatively short amounts of time. This was also impacted by wider (sometimes political) factors, for example, reduced availability of surplus food or increased competition for funding in the third sector^34^ which meant that the way that organisations operated was consistently evolving.

We also found evidence that some objectives produced paradoxical and unintentional consequences. This played out in a variety of ways related to the belief of individual community food organisations about what the underlying objectives of food provision should be and how it should be performed. The ethnographic data was the primary source to understanding the above paradoxical tensions. The community food sector was seen to be highly differentiated in the support it delivered beyond food with some prioritising efficiency, speed and volume as metrics of success, while others valuing building long-term interpersonal relationships and providing meaningful targeted support. This was demonstrated in the ways in which some viewed their core mission as being ‘rapid and high volume suppliers of very cheap or free food’ and others that did not consider food distribution their core ethos or purpose (particularly newer organisations that had organically arisen out of noticing a high population need for support). In one large organisation, researchers observed an average interaction time between volunteers and people accessing the organisation of 3 min. This left little time or space to unpick the wider needs as to why people accessed the organisation.

I asked [two staff members] how they allocate appointments. They said they give five or six families an appointment per 15 minute slot. So that’s three minutes max. per family. In that time, they need to come inside, [staff member] checks if they’re on the IT system, we give them a box, they pay for it, if they’re not on the system they complete a form, then they pack their bags or trolley, ask if they can swap any items they don’t want (or just leave them) and leave the building. It’s a lot to do in a very short time. That’s why there’s pressure to get people in and out of the door quickly and efficiently. And that’s why there isn’t any time for meaningful conversations… (community food organisation 8, fieldnote day 4)

This situation contrasted with that in smaller organisations, where workers/volunteers spent months or years carefully building interpersonal relationships with long-term service users. In many cases, staff actively created the conditions (eg, warm and welcoming waiting area, children’s toys and activities) for people to get to know one another, tackling social isolation and encouraging the organic growth of mutual support. However, the ethnographic data showed that organisations which prioritised slow and careful engagement often had food provision that lacked rationalised processes. This meant the food offering could fall short of meeting people’s everyday needs. For example, the amount and variety of food on offer (as well as the waiting time) were unpredictable and inconsistent, as illustrated in the series of fieldnotes from Community Food Organisation 3:

Most volunteers recognise people when they see them and call them by their first name (Day 2). Many people come back every week, they seem to enjoy spending time together more than anything (Day 3). This site runs children activities and food provision in the same space at the same time. It requires a big number of volunteers, the coordinator is rushed under her feet and looks very tired as a few are missing today (day 4)

Shopping here requires time and demands patience. Some people came before nine and didn’t get to shop until 11:30. The time of the delivery, the number of people available to help sorting the food out of the van and ready to sell impact hugely on the waiting time (day 7).

There is a mismatch between what people want to buy (eggs, milk, tuna) and what gets delivered in overabundance every week (tinned pasta and vegetables). There does not seem to be a mechanism to collect members’ feedback and act accordingly. (day 10)

To sort out the unsafe piling of tins, the pantry coordinator had paused the delivery of non-perishable food. But this means no delivery of tuna, which means that for many (people eating halal food) there will be no suitable meat, fish or egg they can purchase (day 20).

A mother who later stopped using this pantry said in her first interview:

They have fresh stuff, but still there is more earlier and I can’t get there then. And then, they give you tiny amount. And I have a big family. And I tell them: ‘Give me one more, one more, please’. I need to ask everything, even apples, I ask them: “Please, give me one more apple, please”. Because, obviously, I have 4 kids and they all say I want an apple and fight. (parent 2, interview 1)

The availability of welfare or wider support services (eg, employment opportunities) was also deemed to be inconsistent, which meant staff and volunteers did not feel able to ask people about their wider needs. One representative of a national organisation interviewed suggested that ‘there were many people who weren’t maximising their welfare entitlements’. However, this was coupled with capacity, long waiting times, legal and ethical challenges; for example, the risk of ‘inadvertently… giving incorrect advice’, thus necessitating a need for ‘a structure…funding’, staffing investment and insurance (national organisation Y). Where welfare services and wider support were offered in partnership with external agencies, coordination and capacity issues meant that, although available in principle, support was sometimes difficult to access or insufficient. For example a family participant was offered help to apply for a £100 energy bill grant, but there was no capacity to advise (or refer her to an appropriate service) to help her get in touch with the energy provider and discuss the thousands of pounds’ worth of debt accumulated (fieldnotes on third interview with parent 3).

### Can community food organisations play in the prevention of food insecurity in families?

Our data suggested that, although there is potential for community food organisations to provide important support for families (including wider support beyond food aid), prevention of food insecurity was not usually possible in practice. Not only was the delivery of objectives hampered by practical and financial constraints, the influence of wider contextual factors, including those influencing poverty and social security policies, meant that community organisations were most likely to mediate the impact of food insecurity, rather than preventing it. A parent commented:

They are looking at me and saying: come on! We have checked your benefits, we tried to maximise your income… but sometimes you can’t help yourself, sometimes you just need help. […]… But it is good there, overall is good. You get a lot of good-quality food. […] It has become the weekly routine going to the pantry. As soon as I have been down there, I know what we are covered for […] and I am always experimenting with cooking with what we get. […] They are giving help with a lot of things, but there is no other help we can really ask. What we have got, we can’t really change at the minute, we can’t change the structure of how we live. It is about taking it as it is, getting on with it. [Becoming a full time carer] does change your life. (interview with parent 4, interview 1)

Even when they provided services which could play a role in preventing the need for food aid in the future, there were limitations on their actual impact linked to wider structural inequalities, like in the case below, such as the fact that parents with No Recourse to Public Funds due to their migration status cannot access free childcare provision.

They help people to find jobs or do applications. […] They will help to write down a cv and apply for open positions. They helped me to apply for a job in care. But they wanted somebody for full time, but I can only take part-time jobs. My baby is still young. […] There is some help for pregnant women and babies, but I can’t claim it because of my visa restrictions. Sometimes it is quite difficult. My spouse and I try to carefully manage our time. So one can work while one stays with the kids, because paying for a place in a nursery is a lot of money! (parent 5, interview 1)

Our data did not point to a single approach that community organisations use that would be most beneficial to users. Rather, it is more likely that different models cover differing needs. We also found that, rather than any particular approach or model being more or less impactful, the way that support was delivered was perceived to be most influential. In circumstances where organisations had the space and built capacity in staff and volunteers (eg, training, briefing sessions, work on staff/volunteers’ unconscious bias, or through partnerships with external agencies), conversations were able to consider intersecting and longer-term needs, more holistically. This was important, as the majority of people visiting community organisations presented with complex needs that were linked to insufficient income and often with limited opportunities to change their personal circumstances, perhaps due to immigration status, geographical/travel restrictions, caring responsibilities and/or living conditions. We witnessed examples of positive interactions; however, many organisations are overwhelmed due to rising demand and system precarity. As such, dominant activities were often focused on a smooth operating environment to process flows (ref). Some community food organisations focused on distributing food as efficiently as possible, yet they recognised that people’s needs were complex and could not be resolved only through improving access to food:

We want to talk about poverty and people’s experiences of poverty. […] the aim has got to be that people aren’t here and where do we fit in with that? And, that is actually a very complex social piece – it isn’t as simple as giving out food. For some people, giving out that bag of food will mean they can pay a debt off that week […] – it is very significant. For other people, it is the isolation and contact they want. But, others – I have heard a million different reasons why people come and use us. *(*interview with community food organisation 6)

Placing responsibility on community food organisations to reduce food insecurity also ignores the fact that many people are not able to use them (for personal or physical reasons), perhaps highlighting the ethical acceptability of food aid (under what conditions can it be considered as a socially acceptable way to access food?). In our research, families who experienced food insecurity but did not use a community food organisation explained how they associated the idea of using a food bank with accepting a personal failure and had none or very limited knowledge of different models in their area.

Am I really wanting to give the image to someone else that I’m super poor?” Because that’s what we think about when we think about food banks, that they are there to support people who are in dire need. […] I would say it’s almost to the line of shamefulness, although it’s not exactly that, because there’s nothing wrong with getting help when you need it, but I think people in communities, there is a sort of perception or, you know, label you could get stuck with. And once it’s there, it’s very hard to come out of (interview with parent 6, interview 1)

At the same time, many family participants had to confront the social stigma they associated with the idea of entering the building to seek help.

You've got to take that plunge, but it’s hard to take it. I can't even remember how I actually did it, but I did it. And as I said, oh, I have cried the whole thing on. Thinking: “this is embarrassing!”. […] Now I find it more of an ‘hanger’, because coming here gives me that little bit extra money to go and buy nice things for my kids. You know like, take them to a little picnic at the park, you know, just little things like that, make a day special. (interview with parent 7)

Some organisations frequently reflected on the limits of their approach and wished, and in some cases actively campaigned, for wider systemic changes. Only a small minority experimented with cash first approaches; for example, offering small amounts of cash to families in need recently arrived in the area.

I think they need more education, more jobs, if we can give them, rather than only focus on the food and they come here, they only get the food, food, food, food. It’s nice to have extra help. But they need something else. I think the government should pay them rather than them coming here, queueing, waiting. You know, it’s about their dignity, their respect. It’s a lot of things. You're killing yourself, killing your heart. This pack is around £40, £50 worth. So, if that money goes directly to their account, that will benefit. They can buy according to what they need (interview with community food organisation 2)

An asylum-seeking mother commented on her experience of receiving cash:

We are still attending (the community food organisation) where we take the children every Saturday to the English classes. As we arrived, they were giving us £35 as additional help. They called it a voucher, so I was expecting a voucher, but actually it is a small envelope with cash inside. It is money that you can go to claim every month, but it is not always there, we go, but we cannot really count on that because sometimes it is not there. And due to the high cost of transportation we are always considering whether we should or we should not travel to the centre.

[…] you can choose what you really like and want. As they give you cash you have no limitation whatsoever on how you should use it. So you can just go and buy wherever you like whatever you like with your cash. (parent 8, interview 2)

In specific relation to families with food insecurity, free or low-cost food that was reliable and good quality made a difference to families’ everyday lives, but families’ needs were often complex and could not be resolved only through food (eg, housing, mental health, physical health). The places where parents felt most supported were those where there was space to talk, relationships had evolved with staff and other parents over time and staff were attentive to families’ wider needs (eg, school, community centre, community kitchen).

One mother who lived with her husband and their three children told us that both she and her husband were in paid work but on low incomes. Their child’s school set up a food bank during COVID-19, which continued to operate, as well as cooking sessions for parents. It also provided support beyond food, which the mother had accessed for many years:

The school helps a lot. You know [name], she’s very good. She helps a lot. She [organised] courses. Anything, we ask her. They bring children to play. They bring food every Thursday. They do cooking class there. […] I started cooking at the school. Two days [per week], all the parents go there. Every time we cook, we chat. […] The school launched this project, every week they give us food. […] It helps a lot. Because at least if they provide for you the veggies, you can buy the fruit. It reduces the budget. And it helps us to stay healthy. (interview 1 with parent 9)

Families in our research shared how they struggled with rapidly rising prices, including those in paid work. In these circumstances, some families could not access community food organisations because of their working hours. One mother shared that she used to have a well-paid job but, since becoming a single parent, had to move to a role that left her on a low income. She tried accessing a food bank in a children’s centre but supplies had run out:

the line was so gigantic. I think we were ten in a line, and when we reached there everything was gone. […]

Then she tried to join a pantry in a church, unsuccessfully:

Some of my neighbours go there. They have to pay every month, £20, and they get as much food as they can. […] They didn’t have a place, I couldn’t sign up.

And she could not access the food bank at her daughter’s school because she works during the day:

And then [my daughter’s] school as well offers fresh fruit and vegetables for £1, but it’s on [particular day] only, from 2pm until 3pm, so I couldn’t go because I’m working. Pretty sad. (Parent 10, interview 1)

Instead, she managed her budget carefully and creatively, for example, using money-off coupons and apps and shopping in different places for different items.

## Discussion

Throughout the course of this programme of research, we consistently learnt that long-term solutions that address the underlying causes of food insecurity are needed to prevent the need for emergency food. Community organisations play a role to support people experiencing food insecurity among an array of poverty-related issues. Delivered appropriately, they can be welcoming spaces to reduce isolation, provide social connections and signpost to relevant services. They also have the potential to build skills in the community, connect people and increase people’s exposure to a variety of foods and food cultures. However, the fragility of the system and pervasive nature of operations, (alongside a rapid growth in need) often meant that community food organisations got trapped between the need to prioritise the logistics of providing emergency food at scale and addressing the underlying issues of users (reduced time and space to stop and listen). As such, in the current circumstances, we found no evidence that any specific approach was able to prevent the need for emergency food in isolation. Many representatives in this research supported the role of organisations to provide wrap-around services to boost financial resilience, while shifting capabilities and infrastructures and giving ownership back to individuals and/or communities. However, as highlighted by one national community food organisation representative, there are clear issues with ‘the daily ‘tension’ they [community food organisations] wrestle with, about providing the support that people need…whilst influencing so that hopefully they won't need it, whilst not institutionalising ourselves’ (interview with national organisation Z). These tensions have also been highlighted by others, including the Independent Food Aid Network food bank,^35^ which, in an attempt to balance the urgent needs of people, with the desire to find solutions to avoid people’s need to use them, have proposed three pillars; (1) short-term relief (food vouchers, parcels etc), (2) medium-term (signposting, advice) and (3) campaigning to reduce food insecurity (addressing wider drivers of poverty). This is similar to the proposition of primary, secondary and tertiary food support, proposed in our research and suggested by others.^17 18^ Ultimately, these approaches recognise the need for wider systems change to tackle poverty, and thus food insecurity, without ignoring the ongoing needs of people who already need support. Taking this into account, findings from our research have been used to develop and share policy briefs (and recommendations) with national decision-makers (see online supplemental file 3 for an example).

10.1136/bmjopen-2026-122055.supp3 Supplementary data

Other national charities in the UK, including Sustain and the Felix Project have proposed a four principle framework to respond to food insecurity, advocating a cash first approach, providing nutrition security to address dietary inequalities, supporting community food organisations assets (while reducing dependency on emergency food) and implementing good food governance.^36^ Another framework, ‘Food ladders’^37^ offers guidance to build food resilience through supporting community food organisations to provide complementary support to wider government structures from provision of immediate, crisis support (rung 1) to self-organised community initiatives in which communities hold the power to govern and deliver longer term initiatives and resilience food systems (rung 3). Importantly, the role that these envisioned organisations play in delivery of emergency food is negligible, valuing community assets rather than a focus on deficit.

It was clear from our systems mapping work that change needs to consider systems dynamics. This work aligns with others in highlighting that organisation of community food acts as its own system^38 39^ within the wider complex food system.^40^ Relationships with key dependencies in the system (including surplus food, volunteers and external funding) result in an unstable and fragile system that needs to constantly adapt due to external pressures.^5^ Rationalisation of these charitable practices—framed as a choice between ‘Grab and Go’ or ‘Wait and Hope’—fundamentally reshapes how families experience food support, further entrenching a system that operates outside the boundaries of formal rights and dignity. Our work highlights how this notion prevails and, indeed, has expanded since the start of the COVID-19 pandemic. However, an application of stronger governance and policies to support the sustainability of the system does not align with the UK governance strategy to remove the dependency on food aid. In the UK, a new funding model, the ‘Crisis and Resilience Fund’ may offer a step forward to support more longer-term solutions, as this prioritises funding to community food organisations that provide wider ‘preventative’ support.^41^ This funding model was designed with contribution from the community food sector, as well as people with lived experience and it was welcomed by networks campaigning for systemic changes.^42^ Like others, our work suggests that there are potential benefits to users; however, this is unlikely to solve the issue of food insecurity alone.^5^ Moreover, there needs to be further consideration for accountability and monitoring of approaches to ensure that they align and avoid objective drift.

The reliance on surplus food was also an object of debate in this research, as well as in the wider literature.^43^ This argues that the redistribution of surplus food paradoxically reinforces the very issues it aims to resolve, through masking the problem of poverty, allowing industry to frame waste as a ‘charitable donation’ and creating a social divide in access to food (trapping people into relying on surplus food rather than integrating them into a fair, rights-based economy). Additionally, while some participants argued that surplus donations offer an efficient way to prevent food waste and cut cost of waste transport, others argued that, when the quality is low, the cost and effort to dispose of waste is shifted from businesses to community food organisations and, in some cases, to service users.^43^ Other research also suggests surplus food may introduce or heighten stigma^43^ with families noting stress and frustration of using expired or low-quality food.^44 45^ Ultimately, it could be argued that one part of the solution is a more sustainable food system that prioritises human needs, socioeconomic benefits and planetary health.^43 46^

Our work also aligns with others that recognise that reliance on community food organisations, even given ‘ideal’ funding models, cannot appreciably reduce population levels of food insecurity. In addition to wider, economic factors, the community food approach also often comes at an emotional cost. Research both in the UK and internationally supports this, noting that the shame and stigma associated with food are ‘intensified by neoliberal views that frame poverty as a personal failing’.^15 47^ Consequently, individuals often feel they must admit to such failure before being deemed ‘deserving’ of help.^48^ Identifying an appropriate prevention model also poses questions of ethical acceptability of food aid (under what conditions it can be considered as a socially acceptable way to access food) and sustainability of community food organisations, as scholars adopting a ‘right to food’ perspective have previously debated.^49^

While the expansive system of community food may not currently have the ability to prevent food insecurity, our work and the work of others (eg,^11 50^) suggests that they have the potential to provide important spaces for community. Thus, rather than dismantling the existing social capital of such organisations, there may be a way to refine delivery to provide benefits, perhaps via capacity mobilising activities, suggested by the Food Ladders approach^50^; building and maintaining resilience in communities via self-organisation.^51^ Perhaps the new national funding landscape presents an opportunity to debate a core tension: the community food system exists outside of market and welfare structures, resulting in a model based on discretionary assistance rather than a rights-based entitlement. But, as we move towards community/food hubs models, building on community strengths and interdependence and away from deficit models, there may be scope for a reduction in stigma and shame.

### Strength and limitations

This programme of research involved an extensive study describing the community food organisation system and exploring its potential role to support people with food insecurity, with a focus on prevention. We engaged with over 160 actors across the system to ensure we captured the perspectives from a large and varied number of representatives. We included an in-depth ethnography within organisations delivering varying approaches and conducted meaningful and long-term engagement with people with lived experience of food insecurity. While findings have highlighted the potential role played by community food organisations to prevent food insecurity, the lack of quantitative evaluation limits an understanding of the effectiveness of differing models.

## Conclusion

Community food organisations operate in a vast but fragile system, supporting families with multiple complex and often intersectional needs. The majority of them are driven to reduce dependency on emergency food and improve food security. However, up against wider driving factors that influence rising prevalence of poverty, organisations are restricted in their ability to deliver on prevention objectives. Operational tensions are also at play, with a need to deliver highly efficient services to respond to growing demand in tension with the necessity to provide longer-term support with dignity. Newer models prioritise prevention, suggesting that community food systems can drive community cohesion, though are not a solution to food insecurity.

## Supplementary Material

Reviewer comments

Author's manuscript

## Data Availability

Data may be obtained from a third party and are not publicly available. This study includes qualitative data of a very sensitive nature. Participants were informed that their data would not be shared. Other data (not directly collected from families) may be shared under a new data sharing agreement, aligned to our ethical approval.
