# Supplementary material for: Exploring the types of approaches used by community food organisations and their role in food insecurity prevention: a mixed-methods study
Source: BMJ Open. 2026 Oct 22;16(10):e122055. doi: 10.1136/bmjopen-2026-122055 (PMC13711551; doi:10.1136/bmjopen-2026-122055)
Supplement: Supplementary file 1 [file bmjopen-16-10-s001.pdf]

WORK PACKAGE (WP) 1 SCHEDULES/GUIDES

Interview schedule for local Community Food Organisations (WP1)

1. **Background information**: location, services provided, opening hours, when organisation/service was set up, number of staff, number of volunteers, funders, food suppliers.

2. **Aims and logic model/theory of change**:

What are the aims of your organisation/service?

[How does your organisation aim to tackle / prevent food poverty, or poverty more broadly?]

Have your aims and what you do changed in recent years?

Are you planning to make changes? If so, how?

3. **Partnerships and networks:**

a. What are the key organisations you are dependent on in order to operate (locally, regionally, nationally)? And how? (e.g. highly influential/couldn't operate without)
b. Are there any other organisations that you partner with that are beneficial but not essential to your operations? How?
c. Within Bradford / TH can you think of other organisations/services/ people that are essential for allowing people to access the food they need?
d. What are the challenges and opportunities of working in partnership with organisations/services or people?
e. Are you part of a wider network or do you work independently?
f. Are you involved in campaigning at a local, regional or national level?

4. **Target group/s**:

[How] have the needs of your target groups changed over the last few years?

Who might be food insecure but is not accessing your services?

Do you aim to reach these groups? - if so, how?

What barriers are you aware of?

5. **Information and managing access:**

How do people find out about your organisation/service(s)?

How can people access your service(s)?

Is your service open to all, or have you put criteria in place?

6. **Supporting service users:**

How do you identify any underlying needs of people accessing your services?

How do you provide the support they need?

What do you do when you identify needs you can't address?

7. **Access to other services**:

(How) do you know if your service users are also accessing other community food aid organisations, or non-food support services?

Do you feel these are complementary services?

8. **Impact and effectiveness**

What data (if any) do you collect from service users?

What kinds of impact do you measure?

How effective do you believe your service/organisation is in achieving its aims?

To what extent do you believe your service/organisation reduces the need for emergency food? (How?)

What would you say works well?

Who do you share these data with?

Do you evaluate or monitor the impact of your network? (How?)

9. **Challenges:** What are your biggest challenges as a service/organisation providing food assistance and/or other kinds of support?

10. **What needs to change:** What do you feel needs to change locally/regionally/nationally to address the issues underlying food insecurity?

11. **And finally:** Is there anything else you would like to share with me?

Thank you! [explain next steps of the research study, how/when findings will be shared]

<u>**National Organisations - Interview questions WP1**</u>

1. **Activities/services**: Could you describe what your organisation does? What activities and/or services does it carry out with regard to food and related support?

a. Please consider a wide range of activities/services, e.g. types of food support, advice, advocacy, employment and other support, campaigning, policy work.

2. **Type and size of organisation**: How is your organisation structured? Is there a membership? How many staff and volunteers (if relevant) are there? What would you say your organisational 'model' is?

3. Partnerships/networks: Is your organisation part of wider networks, joint campaigns and/or programmes/projects? Could you say a bit about these initiatives if so?

a. How does your organisation work with local initiatives / organisations /services?

b. Can you explain the degree to which your organisation influences local initiatives / organisations / services, or vice versa (e.g. highly influential / slightly influential)
c. Are there any wider organisations or factors that influence the operations of your organisation and if so, to what degree? (e.g. highly influential / slightly influential)
d. Is your organisation is part of a network - can you describe what this is and what the key elements are?
e. If relevant: How are members related to the national organisation? Is there a funding and/or programme/policy relationship? How does this affect the activities/services carried out nationally or by members?

4. **Prevention:** Does your organisation seek to prevent the need for emergency food support, with regard to its activities and/or services? If so, could you describe how this is illustrated in practice? What opportunities and challenges have you faced in this area?

5. **Policy/campaigning:** Is your organisation involved in campaigning and/or policy work at a local, regional and/or national level? If so, what is the broad approach of this work? What changes are you calling for and why? What do you think has been the impact of this work?

6. **Referrals:** Does your organisation (and/or its members) use or operate within referral systems? Does your organisation receive and/or make referrals for people? If so, from/to which organisations/agencies? What types of referrals is your organisation involved in?
a. Does your organisation or its members have criteria in place for people accessing food or related support? If so, what are they and why? (if relevant).
b. What opportunities and challenges has your organisation faced in this area?

7. **Change:** Have your aims and what you do (and those of your members) changed in recent years? Have the needs of your members (if relevant) changed in recent years (e.g. since Covid-19?). Are changes planned? If so, how?

8. **Impact and effectiveness:** What do you think the impact of your organisation has been? How effective do you think your organisation is in working to achieve its aims?).

9. **Challenges:** What are the key challenges faced by your organisation, with regard to achieving its aim(s) and delivering services/activities? (If not already covered).

10. **Supporting service users (if relevant):** How does your organisation and/or its members identify the needs of families or individuals accessing its services? Are tools provided (e.g. training, trained staff) to identify (and address) needs? What happens when the organisations identify needs they can’t address? Do you get involved and how?

11. **What needs to change:** What do you feel needs to change locally/regionally/nationally to address the issues underlying food insecurity?

12. **And finally:** Is there anything else you would like to share with me?

Thank you! [explain next steps of the research study, how/when findings will be shared]
