# Supplementary material for: Exploring the types of approaches used by community food organisations and their role in food insecurity prevention: a mixed-methods study
Source: BMJ Open. 2026 Oct 22;16(10):e122055. doi: 10.1136/bmjopen-2026-122055 (PMC13711551; doi:10.1136/bmjopen-2026-122055)
Supplement: Supplementary file 2 [file bmjopen-16-10-s002.pdf]

WORK PACKAGE (WP) 2 SCHEDULES/GUIDES

Wave 2 topic guide

1. General questions about **wellbeing**.
2. **What's happened in your life** since we last met?
3. **Use of CFO/s** since we last met? Any changes? How do you feel about it?
4. [Discuss **photos** shared by participant.] Can you tell me about this photo? What made you choose to share this with me? Thinking ahead to the photo exhibition next autumn...would you be happy to have any of those photos in the exhibition? if not, which ones you would like to exclude?
5. Thinking ahead to the photo exhibition next autumn... what do you think local or national **policymakers/**the wider public **need to know** about families' access to food? What messages should be shared/conversations could be started through the photos we share in the exhibition?
6. How do you feel about your **ability to access food or living conditions?** - similar/better/worse than when we last met? Can you tell me what has affected/led to this?
7. As part of this research, we're exploring how CFOs might affect families' **health and wellbeing.** Do you have any thoughts about this?
8. How do you feel about the **support provided by the CFO/s** you access? Is there **anything you would change?**
9. **[Any other Qs - things we are curious about following wave 1 interview]**

If need more prompts: school meals, where you shop, prices, changes, eating out...

Wave 3 interviews - questions

Changes over time

1. What has happened in your life since we last met? (inc. changes in use of CFOs)
2. What would you say has been the biggest change in your family's life in the last year (since we met for the first time)?
3. What was the most helpful support you have received in the last year? Where did it come from and how?

4. What was the most frustrating experience of seeking help or advice? Can you tell me what happened?

5. What is the one thing that people working or volunteering in CFOs should know?

6. (Do you ever turn to family or friends for support with food, or other things?)

Photos and photo exhibition

7. How did you choose to share those photos with me? Can you tell what is happening in them?

8. At the exhibition we will have families, CFOs staff and people from the council - what is the key message you would like to share with them through your photos/words?

Reflecting on the process

9. How did you find taking part in this research? What did you like? What you did not like?
