# Supplementary material for: Exploring the types of approaches used by community food organisations and their role in food insecurity prevention: a mixed-methods study
Source: BMJ Open. 2026 Oct 22;16(10):e122055. doi: 10.1136/bmjopen-2026-122055 (PMC13711551; doi:10.1136/bmjopen-2026-122055)
Supplement: Supplementary file 3 [file bmjopen-16-10-s003.pdf]

TACKLING FOOD INSECURITY THROUGH DIFFERENT MODELS OF LOCAL SUPPORT

Dr Rachel Benchekroun, Dr Giorgia Previdoli, Dr Wendy Burton, Professor Bob Doherty, Dr Ariadne Kapetanaki, Dr Philip Hadley, Dr Maddy Power, Professor Maria Bryant and the Fair Food Futures UK Research Team.

Summary

- The food support sector is large and complex. It involves a wide range of organisations such as local authorities, charities, informal groups, education providers and faith groups.
- The food support sector is diverse. Community food organisations (CFOs) include a variety of models, such as food banks, social supermarkets, community cafes, lunch clubs, community kitchens, co-ops, community gardens and soup kitchens. Some CFOs focus on offering food to alleviate food insecurity, others offer a range of services with the aim to reduce/prevent food insecurity and poverty. CFOs also vary in the way they manage eligibility and access to help.
- The majority of CFOs are reliant on an unpredictable supply of surplus food, and/or medium-/short-term funding, including the Household Support Fund (HSF). With the HSF scheduled to end on 30 September 2024, many CFOs are at risk of having to stop or significantly reduce their provision, if alternative funding is not made available by the new government.
- Many CFOs question the logic of the food bank model due to the resource-intensive nature of distributing food; its over-reliance on surplus food donations; and its inability to address the root causes of food insecurity. Some CFOs feel that a 'cash first' approach would be more efficient.

Recommendations for policy

Our findings make clear that CFOs are not a long-term solution to food insecurity and poverty. However, food insecurity remains a significant and immediate societal challenge. Our recommendations therefore address both the immediate and long-term contexts.

1. Coordinate efforts to tackle food insecurity

- Improve coordination by working together as part of wider anti-poverty and sustainable food partnerships.
- Consider a local authority grant funding model prioritising CFOs that focus on strategies to prevent food insecurity, such as welfare rights advice.
- Make funding decisions in a timely way to allow CFOs to plan effectively.
- Support constructive communication between CFOs and surplus food providers to ensure food meets the needs of CFOs and the people who use them.

2. Support CFOs to identify and address people's underlying needs and raise awareness of services

- Support CFOs to provide welcoming, safe spaces where people can disclose wider needs and ask for help.
- Help CFOs to coordinate better with other crisis provision to allow two-way links between them.
- Provide regular training and up-to-date resources to staff and volunteers to ensure they have sound knowledge of other relevant services and know how to signpost and/or refer people to them as appropriate.
- Make it clear exactly how to access food support, both for residents and for professionals.

3. Poverty-proof the system

- To overcome the complexities of the community food support sector, local authorities should consider a cash first approach as piloted in other areas (e.g. Scotland at national level, Leeds at local authority level).
- Short-term relief should not be removed from funding/support as it has a place within the community food support landscape, providing fast, no-strings-attached support to those in emergency need. Other models should also be explored, such as social supermarkets.
- The new government should work towards alleviating food insecurity by reforming the social security system; this includes removing the two-child limit, removing benefit sanctions, and reinstating the £20-a-week uplift to Universal Credit. It should also ensure employers pay a living wage and provide secure employment.

Background

One in five families with children in the UK live in a household where they cannot regularly access affordable and healthy food. Food insecurity has increased significantly as a result of austerity policies since 2010, including the benefit cap and the 'two-child limit'; insecure employment; the expansion of the 'no recourse to public funds' rule; the Covid-19 pandemic; and the cost-of-living crisis. Food insecurity negatively affects physical and mental health. A wide range of CFOs have been set up to provide support.

Research approach

Fair Food Futures UK, a research project funded by the National Institute for Health Research, aims to find out which community food models are most effective in reducing the need for support with food (e.g. food parcels), in two multicultural communities, one in the north and one in the south of England.

In order to build a detailed picture of the community food support system, we carried out a survey of 89 CFOs across Bradford (62) and Tower Hamlets (27) and facilitated two in-person systems mapping workshops in each location (total of 67 participants), including people accessing CFOs. We co-produced typologies and systems maps in online workshops with a further 22 stakeholders. We also interviewed key national and local stakeholders from 36 different organisations, 8 of which were based in Bradford, 14 in Tower Hamlets and 14 worked at a national level.

Findings

Finding 1: Not just food banks! The community food sector is large, diverse and complex

The community food sector at a local level is highly diverse in terms of organisation type, model, aims and approaches to prevention. There are also differences in how organisations manage access, how they work in partnership with others, and access to resources.

Finding 2: CFOs work in different ways to alleviate, reduce or prevent food insecurity

Food support organisations aim to alleviate, reduce or prevent food insecurity and/or poverty more generally. Almost all provide free or low-cost food to people who cannot afford sufficient (or nutritious) food. We call this 'tertiary prevention'. This form of support attempts to address the immediate food need but does not necessarily address the underlying drivers.

Some CFOs address underlying causes by providing advice and advocacy on site or signposting or referring to other organisations ('secondary prevention'). A minority address longer-term needs through education, skills training, volunteering or employment support ('primary prevention').

Finding 3: CFOs face significant challenges

CFOs face a range of challenges. First, there is a reliance on short- or medium-term funding (including the Household Support Fund). Second, there is often a reliance on the redistribution of surplus food. Third, identifying an appropriate prevention model poses questions of ethical acceptability and sustainability. Fourth, it can be difficult to balance the volume of demand with the complexity of people's needs, limited resources and a reliance on volunteers.

Conclusions

Our research highlighted the scale and diversity of the sector; different ways to alleviate, reduce or prevent food insecurity; and challenges and uncertainties in the sector.

Our recommendations offer short-term strategies for local authorities and CFOs, including coordinating provision across local authorities and supporting CFOs to identify and address people's underlying needs. Our long-term recommendations include reform of the social security system and focusing on 'cash first' approaches.

Further information

Read the full report which accompanies this brief.

This brief was researched and written by Fair Food Futures UK, a collaboration among the Department of Health Sciences (University of York), the School for Business & Society (University of York), UCL Social Research Institute, Bradford Institute for Health Research, Tower Hamlets and Bradford Councils. Find out more on https://www.fairfoodfuturesuk.org

This study is funded by the NIHR Public Health Research Programme (NIHR151034). The views expressed are those of the authors and not necessarily those of the NIHR or the Department of Health and Social Care.

DOI number: 10.5281/zenodo.13149830
