# Supplementary material for: Exploring the types of approaches used by community food organisations and their role in food insecurity prevention: a mixed-methods study
Source: BMJ Open. 2026 Oct 22;16(10):e122055. doi: 10.1136/bmjopen-2026-122055 (PMC13711551; doi:10.1136/bmjopen-2026-122055)
Supplement: Supplementary file 5 [file bmjopen-2026-122055.reviewer_comments.pdf]

PEER REVIEW HISTORY

BMJ Open publishes all reviews undertaken for accepted manuscripts. Reviewers are asked to complete a checklist review form and are provided with free text boxes to elaborate on their assessment. These free text comments are reproduced below.

ARTICLE DETAILS

Title (Provisional)

Exploring the types of approaches used by community food organisations and their role in food insecurity prevention: a mixed-methods study

Authors

Bryant, Maria; Previdoli, Giorgia; Benchekroun, Rachel; Burton, Wendy; Power, Maddy; Hadley, Philip; Kapetanaki, Ariadne; Doherty, Bob; Cameron, Claire; Pickett, Kate; Sheard, Laura

---

VERSION 1 - REVIEW

**Reviewer**	**1**
**Name**	**Stoneham, Melissa**
**Affiliation**	**Curtin University, Faculty of Health Sciences**
**Date**	**18-Jun-2026**
**COI**	**None**

Thank you for the manuscript. This is important work and the qualitative nature of your research provided deep data and narratives. I do have some concerns that although your research has adequately identified many challenges, few policy solutions are offered. Food aid is one strategy to address food insecurity. It cannot be seen to be the only solution to the very complex and wicked issue of food insecurity. The complexity of food insecurity was not really addressed in this manuscript and I feel the it would be strengthen if this was acknowledged. I have also attached a document with a number of other suggested amendments.

Please not the pages align with that which is listed at the TOP of each page.

P4– Background

- Add in the UK within the sentence - This is particularly pertinent as food insecurity is greater in some ethnic groups, particularly Black African, Black Caribbean and Black British (22%), Mixed and multiple ethnic groups (20%) and Arab households (21%), compared to White households (8%) (3, 9).

P6 - Setting

- Can the authors provide additional detail about the 2 communities? Goven the manuscript has a specific focus on ethnically diverse and aims to "generate rich evidence to inform culturally appropriate, sustainable community-based policies which could be applied to other diverse populations", some data on evidence to state these communities are multi-ethnic would be useful. Currently there is no reference to this statement "Research was carried out in two areas within the UK; Bradford and Tower Hamlets. Both areas are ethnically diverse with high levels of poverty".

P8 – Intervention

- Can this sentence include references please – "For many organisations, food provision is largely dependent on donations and therefore varies in terms of the offer, its nutritional value and its cultural and/or religious acceptability."

P9 –

- Typo – remove (ref) in line 5, 16 and 21
- Line 33-36 – were all community food agencies in both areas contacted or was it a selected group? Did they all agree? This is important to state to clarify any bias.

P11

- Although go-along interviews are defined here, the use of the term is used earlier in the manuscript. A footnote to provide a description of this method would be useful to accompany the first time the term is used in the paper.

P13

- Can you please identify the 2 communities on P5 in Settings, using the descriptors used here "62 in the <u>Northern site</u> and 27 in the <u>London based site</u>" so readers outside of the UK are aware which community was which.
- Any number below 10 should be spelled out in full – lines 57, 58

P24 - Discussion

- Line 26 - Although not incorrect, it is a well-known fact that food insecurity is a complex, often described as wicked problem where systems approaches are required in order to have any level of change. We know that to address food insecurity we need a multi-faceted approach that integrates short-term relief measures with long-term sustainable strategies. Stating that "Our work suggests that there are potential benefits to users; however, this is unlikely to solve the issue of food insecurity alone. Moreover, there needs to be further consideration for accountability and monitoring of approaches to ensure that they align and

avoid objective drift" seems to imply that your research has only just identified this complexity. I would consider re-wording.

- Line 55 – insert "one part of the solution" - and this line alone is a massive undertaking – what does it really mean in context to your research?

P24

- Line 12 – Covid - capital
- Line 22 – "and the work of others" – please reference these others

There were no suggestions of programs including developing sustainable financing models, including public-private partnerships? Or leveraging AI-driven food waste tracking systems to reduce inefficiencies? Urban agricultural strategies such as the Paris Agripolis Project or London's own Growing Underground project as part of the solutions? I ask because the aim of the research was to provide policy advice on approaches used by community food [aid] organisations that are most likely to help prevent the need for emergency food. Many challenges were identified yet few solutions were. Food aid is one strategy to address food insecurity. It cannot be seen to be the only solution to food insecurity, yet it does play a significant role. The complexity of food insecurity was not really addressed in this research, and I feel the manuscript would be strengthen if this was acknowledged.

Reviewer 2

Name Searle, Aidan

Affiliation National Institute for Health and Care Research, Bristol Biomedical Research Centre

Date 19-Aug-2026

COI None

This manuscript reports on a mixed-methods study exploring community food organisations and their role in food insecurity prevention. The topic area is of high importance and, on the whole, a well conducted, documented and written report but there are a number of points that require further clarification that would enhance the quality and rigour of the manuscript.

Firstly, the reader would benefit from a definition of 'food insecurity' as I believe the term can have different meanings depending on context. For example, is it concerned with food production and distribution or availability, affordability and access for marginalised communities?

More generally, the clarity of the manuscript would benefit from clearer explanation of the integration /synthesis of different data sources and how the themes presented in the results were developed.

With regard to reflexivity - how did the background characteristics of the various researchers affect the extent of candour and richness of the data collected. For example are 'pen portraits' akin to case studies and if so how was this data integrated with the core qualitative data from interview approaches?

With further regard to interview based data were the 'go along' interview questions improvised or was a topic guide followed in an iterative approach? It would be good to have more user/parent representation in the results as there are quotes from just five parents - it would also be useful to know at which interview / timepoint the quotes were taken from. It is noted that there was steep dropout rate following the second interviews - what is this attributed too and what efforts were made for participant retention?

In addition, how did the different data collection methods fare with regard to their relative contribution to the findings - it would be appropriate to appraise the methods as 'strengths and limitations' of the study methodology in the Discussion.

Finally, an evaluation of the methodology would be useful with regard to future research both within and beyond the context of the present manuscript.

VERSION 1 - AUTHOR RESPONSE

	**Reviewer 1**	
R1	Thank you for the manuscript. This is important work and the qualitative nature of your research provided deep data and narratives. I do have some concerns that although your research has adequately identified many challenges, few policy solutions are offered. Food aid is one strategy to address food insecurity. It cannot be	Thank you for this overarching comment. Our paper and study focuses specifically on the role of community food organisations in addressing food insecurity in two cities. To maintain our focus of answering our original NIHR funded research aims and specifications, we do not use this paper to discuss the complexity of food insecurity itself. Thus, rather than providing policy solutions to food insecurity, our intention is to significantly advance the evidence base on the diversity, strengths and limitations of community food aid. Alongside this paper, we have also provided detailed policy recommendations within national and local government policy briefings. A supplementary file (3)

	seen to be the only solution to the very complex and wicked issue of food insecurity. The complexity of food insecurity was not really addressed in this manuscript and I feel the it would be strengthen if this was acknowledged.	these has now been provided in the paper. These recommendations align with well-established knowledge from academic and third sector communities of the necessary policy responses to food insecurity (e.g. changes to various aspects to social security, such as Trussell's Essentials Guarantee (collaboration with LSHTM: https://www.trussell.org.uk/news-and-research/publications/report/living-costs-in-context).<br><br>Our conclusion also highlights how the work is positioned within a fragile system, supporting families with multiple, complex and often intersectional needs.
R2	P4– Background<br>• Add in the UK within the sentence - This is particularly pertinent as food insecurity is greater in some ethnic groups, particularly Black African, Black Caribbean and Black British (22%), Mixed and multiple ethnic groups (20%) and Arab households (21%), compared to White households (8%) (3, 9).	Added to amended text (P.7)
R3	P6 - Setting<br>• Can the authors provide additional detail about the 2 communities? Given the manuscript has a specific focus on ethnically diverse and aims to “generate rich evidence to inform culturally appropriate, sustainable community-based policies which could be applied to other diverse populations”, some data on evidence to state these communities are multi-ethnic would be useful. Currently there is no reference to this statement	Additional text and a reference has been added (p. 9)

	"Research was carried out in<br>two areas within the UK; Bradford and Tower Hamlets. Both areas are ethnically diverse with high levels of poverty".	
R4	P8 – Intervention<br>• Can this sentence include references please – "For many organisations, food<br>provision is largely dependent on donations and therefore varies in terms of<br>the offer, its nutritional value and its cultural and/or religious acceptability."	A new reference (Oldroyd 2022) has been added. This has been moved to the Introduction, based on Editorial review (p.4)
R5	P9 –<br>• Typo – remove (ref) in line 5, 16 and 21<br><br>• Line 33-36 – were all community food agencies in both areas contacted or was it a selected group? Did they all agree? This is important to state to clarify any bias.	'Ref' removed.<br><br>Yes during our systems mapping exercise, all community food organisations in both areas were contacted. Text to describe this process has been added to p. 10
R6	P11<br>• Although go-along interviews are defined here, the use of the term is used<br>earlier in the manuscript. A footnote to provide a description of this method would be useful to accompany the first time the term is used in the paper.	We have added text the first time 'go-along interviews' are mentioned (not including the abstract) to refer to the detailed section on go-along interviews as " (see 'Go-along interviews' below). (p.8)
R7	P13<br>• Can you please identify the 2 communities on P5 in Settings, using the descriptors used here "62 in the Northern site and 27 in the London based	We have added text describing the localities with 'Setting' (p9).<br><br>Numbers under 10 are now written as text

	site" so readers outside of the UK are aware which community was which.<br>• Any number below 10 should be spelled out in full – lines 57, 58	
R8	P24 - Discussion<br>• Line 26 - Although not incorrect, it is a well-known fact that food insecurity is a complex, often described as wicked problem where systems approaches are required in order to have any level of change. We know that to address food insecurity we need a multi-faceted approach that integrates short-term relief measures with long-term sustainable strategies. Stating that "Our work suggests that there are potential benefits to users; however, this is unlikely to solve the issue of food insecurity alone. Moreover, there needs to be further consideration for accountability and monitoring of approaches to ensure that they align and avoid objective drift" seems to imply that your research has only just identified this complexity. I would consider re-wording.<br><br>• Line 55 – insert "one part of the solution" - and this line alone is a massive undertaking – what does it really mean in context to your research?	The reviewer is correct in that this work follows a body of similar literature. We feel that our work builds upon this and have added text and references to highlight how our findings aligns to that of others. (p. 30)<br><br>"One part of the" has been added as suggested. (p.31)
R9	P24<br>• Line 12 – Covid - capital<br>• Line 22 – "and the work of others" – please reference these others	Covid capitalised<br>Additional papers now included to highlight how our findings align with previous research.

R10	There were no suggestions of programs including developing sustainable financing models, including public-private partnerships? Or leveraging AI-driven food waste tracking systems to reduce inefficiencies? Urban agricultural strategies such as the Paris Agripolis Project or London's own Growing Underground project as part of the solutions? I ask because the aim of the research was to provide policy advice on approaches used by community food [aid] organisations that are most likely to help prevent the need for emergency food. Many challenges were identified yet few solutions were. Food aid is one strategy to address food insecurity. It cannot be seen to be the only solution to food insecurity, yet it does play a significant role. The complexity of food insecurity was not really addressed in this research, and I feel the manuscript would be strengthen if this was acknowledged.	The specified focus of this paper is an exploration of the role of community food organisation in addressing food insecurity in two cities. The organisations that took part in the study were mainly those at the sharp end who provided emergency assistance such as food banks or food pantries. We use the Discussion section of our paper to discuss the findings in context with the extant literature and policy *as related to the types of organisation involved in the study*. As such, solutions such as AI driven waste tracking systems and urban agricultural strategies are not in keeping with the findings of what workers/volunteers at the study organisations told us.(i.e. they were not considered as viable solutions to solve the immediate problem of hunger and over-demand on their services given the constant state of intense firefighting they perform everyday).<br><br>We did not intend to use our paper to discuss the complexity of food insecurity (see response to first comment) or call for high level solutions to food insecurity as these types of solutions are already well informed by the existing academic literature. In addition, we call attention to the complexity of food insecurity as observed in our study in a different paper currently in press. We focus in this current paper on solutions proposed by our participants and partner organisations.
	**Reviewer 2**	
R11	Firstly, the reader would benefit from a definition of 'food insecurity' as I believe the term can have different meanings depending on context. For example, is it concerned with food production and	We have added the WHO definition of food security to the start of the introduction.

	distribution or availability, affordability and access for marginalised communities?	
R12	More generally, the clarity of the manuscript would benefit from clearer explanation of the integration /synthesis of different data sources and how the themes presented in the results were developed.	Thank you. Your comment has made us realise that the manuscript as it stands did not contain a distinct section in the Methods regarding how the different data sources were analysed and integrated. We have now added a new section into the paper (p.15-16) to improve our reporting of the analytic process we undertook. The content we have added is detailed below:<br><br>*The interviews and workshop transcripts from the systems mapping were coded line by line. The field notes from ethnographic observations and the interview transcripts with parents were distilled into pen portraits (see response to Point 14). The analytic integration was led with a focus on answering our core funded research questions. These were: 1) what does the community food support system look like, across two cities? 2) can community food organisations support people beyond and, if so, how? 3) do community food organisations have a role in preventing food insecurity for families and, if so, in what way?*<br><br>*We followed an iterative process to enhance rigour: first, the lead author engaged with the data and other material such as team presentations then second, the lead author engaged in reflexive conversations with the researchers who collected the data in the field. Finally the lead author and the researchers who had collected the data drafted sections of findings under the headings of the three core research questions and shared the content with the wider team for feedback and suggestions.*
R13	With regard to reflexivity - how did the background characteristics of the various researchers affect the extent of candour and richness of the data collected.	The following text has been added to page 15:<br><br>*The ethnographic researchers reflected throughout the research process (from fieldwork to analysis and writing) on how different aspects of their identities influenced how they interacted and built relationships with participants, how data was generated and how they analysed it. They regularly reflected as a team on how their gender, motherhood status, migration/citizenship statuses, religious beliefs and researcher roles shaped the research, and reflexively discussed their own assumptions and perspectives. For example, they felt that being mothers themselves helped to connect with participants, all of whom had children under 18. Participants' willingness to discuss*

		their experiences will have varied depending on their circumstances at the time as well as the rapport they felt with the researchers.
R14	For example are 'pen portraits' akin to case studies and if so how was this data integrated with the core qualitative data from interview approaches?	Pen portraits (Sheard & Marsh, 2019) are a qualitative analytic technique developed to bring together a volume of qualitative data and focus on understanding change over time. They are not strictly akin to case study analysis, which usually takes a much less structured approach. Pen portraits were produced for each of the nine community food organisation sites and for each of the families that took part in 'go along' interviews. Thank you for pointing out that it is not clear how the pen portraits from the two data sources were integrated during the analysis stage. We have added more detail on this process (bottom of page 16)
R15	With further regard to interview based data were the 'go along' interview questions improvised or was a topic guide followed in an iterative approach?	The first wave of go-along interviews were designed to be open-ended and participant-led to build a relationship between researcher and participant and to understand what issues were important to participants. For waves 2 and 3 of the go along interviews, we designed topic guides which included topics for the researchers to ask participants about, but remained participant-led. We have added this information to the manuscript (p.16) and have included the topic guides to the supplementary materials (supplementary files 1 and 2).
R16	It would be good to have more user/parent representation in the results as there are quotes from just five parents.	We have added into the paper further quotes from 5 more parents (bringing the total to 10 parents altogether).
R17	It would also be useful to know at which interview / timepoint the quotes were taken from.	Yes we agree and note this omission. We have now added this timepoint information into the paper.
R18	It is noted that there was steep dropout rate following the second interviews - what is this attributed too and what efforts were made for participant retention?	We anticipated a high drop out rate for family participants in this study due to the busy nature of people's lives with many conflicting priorities. At the outset, we believed the drop out rate would actually be higher. Forty families were interviewed in the first round of interviewing and this had dropped to 30 families by the third round, representing an attrition rate of 25%. Drop out rate rules of thumb in longitudinal interview studies are around 40 to 50% on

		average. Higher than average participant retention was maintained by the researchers developing strong and trusting rapport with participants.
R19	In addition, how did the different data collection methods fare with regard to their relative contribution to the findings - it would be appropriate to appraise the methods as 'strengths and limitations' of the study methodology in the Discussion.	We have not sought to assess methods according to their relative contribution to the findings. In interpretative and especially ethnographic research, different data sources are not necessarily expected to make equivalent contributions towards the analysis nor it is always warranted that all participant groups should be represented equally.<br><br>Sheard (2022) has noted that in interpretative understandings of qualitative research, weighting of different data collection methods and therefore trying to represent all methods and participants equally can constitute a false obligation. That is, qualitative representation does not ensure methodological rigour.
R20	Finally, an evaluation of the methodology would be useful with regard to future research both within and beyond the context of the present manuscript.	Yes, thank you for this. Two of the researchers are currently writing a methodological article which explores the challenges for ethnographic researchers navigating multiple roles in the field (volunteer, researcher, advocate…).

VERSION 2 - REVIEW

**Reviewer**	**1**
**Name**	**Stoneham, Melissa**
**Affiliation**	**Curtin University, Faculty of Health Sciences**
**Date**	**29-Sep-2026**
**COI**	

Thank you for addressing the issues raised. This is now a strong manuscript.
